# Malnutrition, protein energy wasting and sarcopenia in patients attending a haemodialysis centre in sub-Saharan Africa

**DOI:** 10.1038/s41430-024-01458-0

**Published:** 2024-06-12

**Authors:** Findlay Crystal, Robert Fulai, Patrick Kaonga, Andrew Davenport

**Affiliations:** 1 UCL Division of Medicine, University College London, London, UK; 2 Department of Internal Medicine, University Teaching Adult Hospital, Lusaka, Zambia; 3 Department of Epidemiology and Biostatistics, School of Public Health, University of Zambia, Lusaka, Zambia; 4 Department of International Health, Bloomberg School of Public Health, Johns Hopkins University, Baltimore, MD USA; 5 UCL Centre for Kidney & Bladder Health, Royal Free Hospital, University College London, London, UK

**Keywords:** Acid, base, fluid, electrolyte disorders, Nutrition, Skeletal muscle

## Abstract

**Background:**

Haemodialysis (HD) patients are reported to be at greater risk of malnourishment, and at risk of increased morbidity and mortality. However, most studies report from economically advanced countries. We therefore assessed the nutritional status and diet among HD patients attending a public university hospital in a sub-Saharan African country.

**Subjects:**

We performed nutritional assessments in HD patients attending the largest dialysis centre, in the country, collecting demographic and clinical data, dietary intake, along with anthropometric and bioimpedance body composition measurements in May 2022. Malnutrition was classified according to subjective global assessment score (SGA). Additional assessments of protein energy wasting (PEW), clinical frailty, and sarcopenia were made.

**Results:**

All 97 HD patients were recruited, mean age 44.7 ± 12.2 years, with 55 (56.7%) males. Malnutrition was present in 43.8%, PEW 20.6%, frailty 17.6% and sarcopenia 4.1%. On multivariable logistic regression higher serum albumin (adjusted odds ratio (AOR) 0.89, 95% confidence intervals (CI) 0.85-0.95, *p* < 0.001), creatinine (AOR 0.99, 95%CI 0.98–0.99, *p* < 0.001), greater mid upper arm circumference (AOR 0.89, 95%CI 0.83–0.95, *p* = 0.001), body cell mass (BCM) (AOR 0.79, 95%CI 0.67–0.95, *p* = 0.013) and employment (AOR 0.45, 95%CI 0.23–0.87, *p* = 0.017), were are all protective against malnourishment. Almost 75% had reduced dietary protein intake.

**Conclusions:**

Despite a younger, less co-morbid patient population, malnutrition is common in this resource poor setting. The staple diet is based on maize, a low protein foodstuff. Employment improved finances and potentially allows better nutrition. Further studies are required to determine whether additional dietary protein can reduce the prevalence of malnutrition in this population.

## Introduction

Chronic kidney disease (CKD) is now one of the most important non-communicable diseases world-wide. Prior to starting dialysis patients may be treated with low protein diets, and even after starting dialysis patients have restricted diets designed to limit sodium, potassium and phosphate intake [1, 2]. As such dialysis patients are at increased risk of malnutrition [3].

In economically developed countries the number of patients treated by dialysis, particularly haemodialysis (HD), continues to increase annually, with the greatest exponential increase being the number of the elderly patients starting dialysis. Muscle mass naturally declines with age, but a greater loss of muscle mass than that expected for age, termed sarcopenia, is associated with increased risk of mortality both in geriatric and HD populations [4, 5]. The European Working Group for Sarcopenia in Older People (EWGSOP) and Foundations for the National Institute of Health (FNIH) have developed criteria for the assessment of sarcopenia based on non-invasive measurements of muscle mass using various techniques including anthropometry, bioimpedance, dual-energy x-ray absorptiometry, and functional assessments of muscle strength or performance [6]. World-wide, the subjective global assessment (SGA) is the tool most used by dietitians to assess the nutritional status of dialysis patients [7]. The combination of malnutrition and muscle wasting is often termed protein energy wasting (PEW) [5].

As dialysis is now offered to more older patients with advanced CKD, and those with additional co-morbidities in economically advanced countries, then more patients with degrees of frailty are now receiving dialysis [8]. Frail patients tend to be less physically active and have lower muscle mass [9, 10] and are greater risk of both sarcopenia and PEW [10]. In clinical practice frailty can be easily assessed using the 9-point Clinical Frailty Scale (CFS) [11], and frail HD patients have been reported to be at increased risk of both hospitalisation and mortality [12].

The prevalence of sarcopenia reported in dialysis patients varies between studies [11–15], but these studies have predominantly reported patients dialysing in Western Europe and North America, whereas there are very few reports from dialysis centres in developing countries [15]. As such we wished to investigate the prevalence of malnutrition, sarcopenia, and frailty in patients dialysing in a centre in sub-Saharan Africa.

## Methods

### Patients

A nutritional assessment of adult patients attending for HD at a university hospital in sub-Saharan Africa was undertaken during May 2022. All patients dialysed for 4 h thrice weekly, and only those established on HD for more than 3 months were included in the study.

### Methods

Post-dialysis weight (kg)/height (m)^2^ was used to calculate body mass index (BMI). Weight was measured with a calibrated scale, and height using a stadiometer. Anthropometric measurements were obtained by the hospital nutritionist, dialysis nurse, or principal investigator. The mid-upper arm circumference (MUAC) was measured to the closest 0.1 cm in the non-fistula arm with the patient standing and arms hanging down [16]. Hand grip strength (HGS) was measured with a Takei digital dynamometer (Takei Scientific Instruments, Shinagawa-Ku, Tokyo) using a standardised protocol [17]. Normal blood pressure was considered when systolic was less than 120 mmHg and diastolic less than 80 mmHg. The majority of blood tests were measured at a private diagnostic laboratory. Dialysis adequacy was calculated using the Daugirdas equation and normalised protein nitrogen accumulation (nPNA) using: nPNA = 0.22 + (0.036 * intradialytic rise in BUN * 24)/(intradialytic interval) [18]. Malnutrition was assessed using the 7-point SGA; with values of 6–7 = very mild risk/normal nutritional status, 3–5 = mild/moderate malnutrition and 1–2 = severe malnutrition [19]. In addition, body composition was measured using the Body Composition Monitor (Fresenius AG, Bad Homberg, Germany), with measurements made 20 min after dialysis with electrodes placed on the non-fistula arm and leg [20, 21]. Demographics, and relevant co-morbidity were obtained from the hospital health care records, and patients assessed for clinical frailty using the clinical frailty score (CFS) [11].

### Ethics

This observational, cross-sectional study was approved by the local university ethics committee. All patients provided written informed consent in keeping with the principles of Helsinki. Patients were told they were free to withdraw from the study or skip any questions without any consequences. Out of the 97 participants we approached, everyone agreed to take part in the study.

### Statistical analysis

Data was checked for normality using the Shapiro-Wilk test, and expressed as mean and standard deviation, or median and interquartile range as appropriate. The Chi square (X^2^) test was used for analysis of categorical data. Bonferroni post hoc adjustments were made as appropriate. Cohen’s kappa statistic was used to compare the different scoring tools for assessing malnourishment, frailty, and sarcopenia. As only 2 patients had SGA scores of 2 or less, we combined severely and moderately malnourished patients to develop a multivariable binary logistic regression model to determine factors associated with malnutrition. Variables with a univariate *p* < 0.05 were included into a step backward regression model, which was checked for collinearity and variance inflation factor (VIF). All analyses were made using SPSS version 24 (IBM SPSS Corp., Armonk, New York, USA). Statistical significance was taken with a *p* < 0.05.

## Results

We assessed the nutrition status of all 97 eligible patients 44.7 ± 12.2 years, 55 (56.7%) male. The majority (81.4%) were from within the capital city; 63.8% were married, 59.6% unemployed, 50.5% tertiary education, 75% had hypertension and 37.6% were human immunodeficiency virus (HIV) positive. Approximately three-quarters (74.1%) of participants had inadequate protein intake (nPNA <0.8 g/kg/day).

Thirty-nine patients (43.8%) were malnourished using the SGA assessment and they had significantly lower MUAC, HGS, body cell mass (BCM), lean tissue index (LTI), serum albumin, and creatinine compared (Table 1). Malnourished patients were shorter, but this difference did not remain statistically different after Bonferroni adjustment.

**Table 1:** Baseline and clinical characteristics of study participants.

Characteristic	Not malnourished	Malnourished	*P* value
number	50	39	
Age in years, mean (SD)	44.7 (11.8)	43.5 (13.1)	0.635
Sex (*n*, %)			
Male	33 (66.0)	18 (46.2)	0.060
Female	17 (34.0)	21 (53.8)	
Residence (*n*, %)			
Lusaka	40 (80.0)	33 (84.6)	0.574
Outside Lusaka	10 (20.0)	6 (15.4)	
Marital status (*n*, %)			
Married	30 (62.5)	25 (64.1)	0.877
Not married	18 (37.5)	14 (35.9)	
Employment status (*n*, %)			
Employed	25 (50.0)	10 (27.0)	0.031
Not employed	25 (50.0)	27 (73.0)	
Education level (*n*, %)			
No education/primary	18 (20.2)	9 (23.1)	0.509
Secondary	13 (26.0)	13 (33.3)	
Tertiary	28 (56.0)	17 (43.6)	
Blood Pressure (*n*, %)			
Normal	8 (18.2)	11 (30.6)	0.196
High	36 (81.8)	25 (69.4)	
HIV status (*n*, %)			
Negative	32 (68.1)	25 (61.5)	0.526
Positive	15 (31.9)	15 (38.5)	
Protein Intake (nPNA)			
Adequate	11 (22.0)	11 (29.7)	0.477
Inadequate	37 (77.1)	26 (70.3)	
Height (cm)	166.4 (9.0)	162.3 (8.3)	0.029
BMI kg/m^2^	22.9 (20.7–24.7)	23.0 (20.1–25.0)	0.697
MUAC post-dialysis	24.9 (23.0–26.8)	22.5 (19.6–25.0)	0.006
Hand Grip strength kg	22.3 (19.9–30.0)	17.4 (12.3–21.9)	<0.001
LTMI in kg/m^2^	12.1 (10.7–13.4)	10.6 (9.0–12.7)	0.036
BCM kg/m^2^	18.0 (5.3)	15.4 (6.1)	0.031
TBW in L	30.6 (27.3–35.4)	29.8 (23.9–31.9)	0.141
Overhydration in L	2.0 (0.2–3.6)	2.3 (0.6–4.7)	0.179
ECW/ICW	0.9 (0.8–1.1)	1.0 (0.9–1.2)	0.125
ECW in L	15.4 (3.0)	14.9 (3.1)	0.471
FTMI kg/m^2^	9.7 (7.0–13.9)	10.5 (7.7–15.0)	0.482
Haemoglobin g/dL	8.9 (8.0–10.9)	8.4 (7.1–10.6)	0.295
Albumin g/L	38.2 (4.0)	34.4 (5.4)	<0.001
Total serum protein g/L	69.3 (8.9)	67.2 (8.9)	0.294
Serum creatinine µmol/L	961.2 (752.9–1112.0)	809.1 (757.6–1097.2)	0.040
Serum urea mmol/L	7.3 (5.9–9.0)	6.3 (4.6–10.5)	0.461
Dialysis vintage months	34.0 (17.0–48.0)	21.0 (68.0–46.5)	0.050
Kt/Vurea	1.18 (0.89–1.42)	1.37 (1.10–1.77)	0.054

Normal nutritional assessment SGA ≥ 6, malnourished SGA ≤ 5, blood pressure, human immunodeficiency virus (HIV), body mass index (BMI), mid upper arm circumference (MUAC), lean tissue mass index (LTI), body cell mass (BCM), total body water (TBW) fat tissue mass index (FTI), normalised nitrogen protein accumulation (nPNA), dialysis adequacy (Kt/Vurea). Data expressed as mean ± SD or median (IQR). *P* values comparing those with normal nutritional assessment and those malnourished.

*SD* standard deviation, *HIV* human immunodeficiency virus, *BMI* body mass index, *MUAC* mid upper arm circumference, *IQR* interquartile range, *LTI* lean tissue index, *BCM* body composition monitoring, *FTI* fat tissue index, *Kt/V* represents the dose of haemodialyses, an abbreviation of (KureaTd)/Vurea. Kurea (mL/min).

**Fig. 1:**
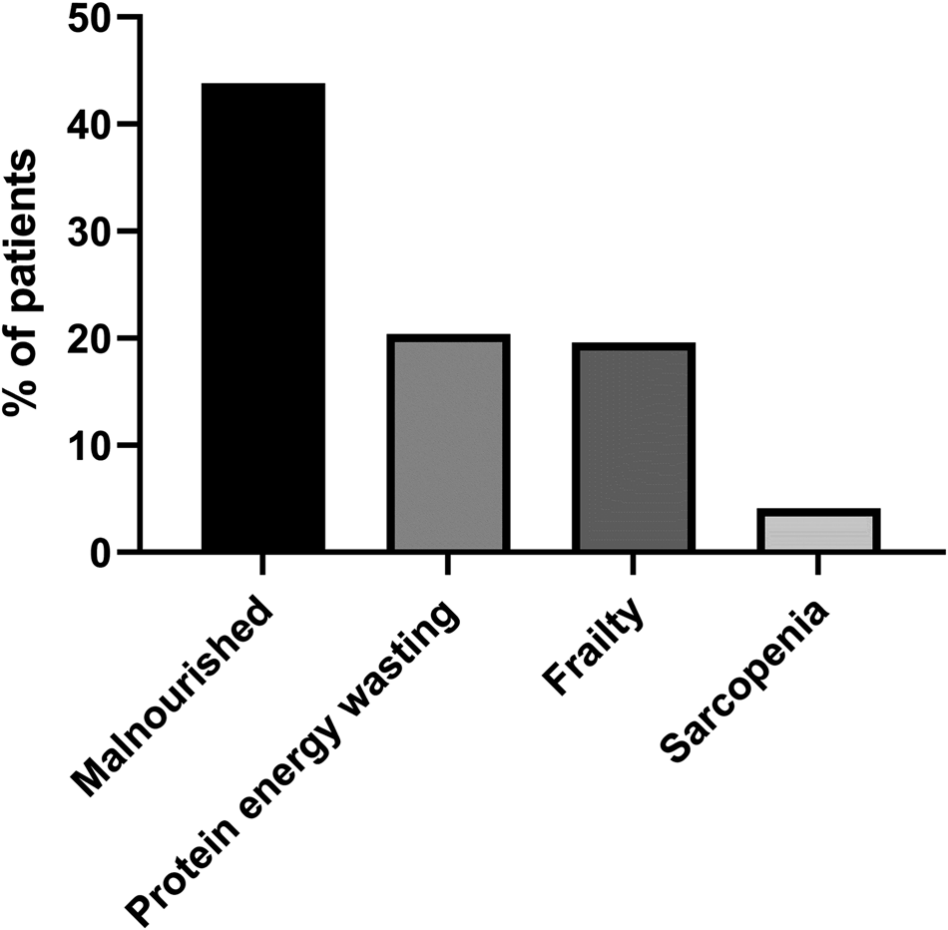
Prevalence of malnutrition based on four nutritional screening tools.

Using the CFS [11], then 19 (19.6%) were classified as frail, and 20 (20.4%) had PEW according to the International Society for Renal Nutrition and Metabolism (ISRNM) criteria for PEW [15], and 4(4.1%) met the EWGSOP definition of sarcopenia (Fig. 1). All 97 patients were assessed for frailty and had HGS and bioimpedance measured to screen for frailty, MUAC measurements were made in 89 (92%) patients for the 7-point SGA, and relevant blood biochemistry tests were available for 82 (85%) patients as part of the screen for PEW.

Kappa analysis was used to assess the agreement among nutritional assessment tools used. The kappa statistic can take values from −1 to 1. The agreement between SGA and sarcopenia were 0.09 suggesting agreement equivalent to chance; SGA and frailty was 0.22 suggesting fair agreement; SGA and PEW were 0.15 which is considered as slight agreement: sarcopenia and frailty were 0.21; sarcopenia and PEW were -0.02, while frailty and PEW were 0.06 (Table 2).

**Table 2:** Agreement among different malnutrition assessment tools: analysis by Cohen’s kappa statistic. Subjective global assessment (SGA), protein energy wasting (PEW).

Tool	SGA	Sarcopenia	Frailty	PEW
SGA	1	0.09	0.22	0.15
Sarcopenia	0.09	1	0.21	−0.02
Frailty	0.22	0.21	1	0.06
PEW	0.15	−0.02	0.06	1

On multivariable logistic regression analysis, malnourishment was associated with a lower serum albumin, creatinine, body cell mass, and MUAC (Table 3). Patients who were in employment were 55% less likely to be malnourished (AOR = 0.45; 95% CI: 0.23–0.87). On average, one unit increase in creatinine was associated with a 1% decrease in the odds of being malnourished (AOR = 0.99; 95% CI: 0.98–0.99).

**Table 3:** Factors associated with malnutrition.

Characteristic	AOR	95% CI	*P* value
Employment status			
Unemployed	Ref.		
Employed	0.45	0.23–0.87	0.017
albumin g/L	0.89	0.85–0.95	<0.001
creatinine umol/L	0.99	0.98–0.99	<0.001
MUAC cm	0.89	0.83–0.95	0.001
LTMI kg/m^2^	1.11	0.98–1.25	0.092
BCM kg	0.79	0.67–0.95	0.013

Step backward multivariable logistic regression model, which was checked for collinearity and variance inflation factor. Adjusted odds ratio (AOR), 95% confidence intervals (95% CI), mid upper arm circumference (MUAC), lean tissue mass index (LTMI), body cell mass (BCM). Factors with an AOR of <1.0 are associated with no malnutrition.r2 = 0.23.

*AOR* adjusted odds ratio, *CI* confidence interval, *LTMI* lean tissue mass index, *BCM* body cell mass.

## Discussion

Most studies reporting on nutritional assessments in dialysis patients come from economically developed countries [15]. We therefore report on adult patients dialysing at the largest tertiary hospital in the country and the only public hospital with a specialised renal unit in the country. Almost 75% of patients had reduced dietary protein intake, when compared to that advised by clinical guideline committees [3, 19]. The prevalence of malnutrition was 43.8% using the 7-point SGA assessment [19], and malnutrition was independently associated with lower serum albumin, creatinine, MUAC, BCM and unemployment. The prevalence of PEW and sarcopenia were lower, being 20.4% and 4.1%, respectively and 19.6% classified as frail using the CFS [11, 15, 22]. The 7-point SGA includes assessment of weight change, dietary intake, gastrointestinal symptoms, functional ability, co-existing co-morbidity, and physical examination. Whereas there was fair agreement between SGA and frailty, there was only slight agreement with PEW and no agreement with sarcopenia. As our patient cohort was younger and had fewer co-morbidities than those typically dialysing in economically advanced countries, this may have impacted on SGA scores. In addition, we used the cut-offs from European and North American clinical guidelines to screen for sarcopenia, and these may not be appropriate in a sub-Saharan African population, and may account for the poor association between SGA and sarcopenia.

Compared to other studies, the reported prevalence of malnutrition in our study was lower than that reported in other studies [15]. One study from Egypt reported a much higher prevalence of 85% [23], and one from Nigeria 55% [24]. The difference in prevalence between these studies published almost 10 years ago, could reflect differences in terms of access to dialysis, as patients may have to pay in full or part for dialysis treatments in developing countries, so having less than thrice weekly sessions and re-using low-flux dialyzers, along with differences in comorbidities, dialysis vintage, let alone dietary habits [25], whereas all the patients we report were in receipt of what would now be considered standard of care with thrice weekly 4-hour dialysis sessions.

Failure to achieve adequate clearance of uraemic toxins has been reported to increase the risk of PEW [26]. All our patients dialysed for 4 h thrice weekly, even so only 55% achieved a sessional KT/Vurea target of ≥1.4, and there was no associated between sessional Kt/Vurea and SGA scores, which supports a previous report from Iran which also reported no association between dialysis session urea clearance and nutritional status [27].

Serum albumin can be lowered by inflammation, PEW and so not just a marker of malnutrition, and the mean serum albumin was below the ISRNM advisory target level of 38 g/L in our patient cohort [28]. However, whether our patients were classified as malnourished by SGA criteria, or those with PEW, then all had a low serum albumin [15, 19]. Those classified as frail had a mean lower albumin than those who were not frail though the result was not significant [11]. The number of patients who were classified as sarcopenic was low (*n* = 4), therefore, due to this no further analysis was done.

Similarly, serum creatinine was lower in our patients who were malnourished, which is in keeping with reports from Turkey [29], demonstrating the association between lower serum creatinine in dialysis patients and reduced muscle mass and malnutrition. For both frailty and PEW, patients who were malnourished had lower values compared to those who were not malnourished. For frailty, the result was not statistically significant, but for PEW, there were significant differences.

However, another observational study from Iran found no association between serum creatinine and malnutrition [30]. Although this study did show a significant difference in the prevalence of malnutrition between male and female patients, with greater moderate malnutrition observed with male patients, which have biased the study results. Creatinine is generated from muscle creatine, so more physically active patients will generate more creatinine. In our study patients who were employed were less likely to be malnourished, and this is in keeping with other reports of physical activity linked to employment, and reduced prevalence of PEW, sarcopenia, and frailty [9]. Our malnourished patients had lower body cell mass and more importantly lower lean tissue when indexed for height, in keeping with less muscle mass. Similarly, the MUAC was lower in our malnourished patients, and as there was no difference in fat mass indexed for height, this would again suggest lower upper arm muscle mass in the malnourished patients.

Creatinine is also affected by diet, in particular dietary meat protein intake. We found that the median nPNA value was well below the 2020 Kidney Disease Outcomes Quality Initiative (KDOQI) Clinical Practice Guideline for Nutrition recommended dietary protein intake 1.0–1.2 g/kg/day [3], and almost three-quarters (74%) of the patients had low protein intake (nPNA < 0.8 g/kg/day) which could be affected by diet patterns. The primary staple food is a starch-based food (maize) and as most individuals, especially those from poorer households predominantly only eat maize with only very little meat, this could explain the lower nPNA reported in our study [31]. Although there was no significant difference in the dietary protein intake in those who were malnourished and not malnourished, this may have been confounded by all patients being given a meal when they attended for their dialysis session. As such most dietary restrictions and recommendations for dialysis patients developed for economically advanced countries [1, 3], may be inappropriate for patients living in sub-Saharan Africa. Therefore, it is important that dietary recommendations should be appropriate for the dialysis population, considering geopolitical, religious, and other factors, including ethnicity. Whereas in economically developed countries emphasis on protein and phosphate restriction may be appropriate [3, 4], in resource-limited settings in developing countries more attention is required to provide dietary advice to ensure adequate nutrition. Our unemployed patients were more likely to be malnourished, and economic factors, such as the lack of financial resource to purchase essential foods may have played a role in the development of malnutrition. In our study, a higher proportion of individuals who were unemployed had low nPNA, though when compared to those who were employed, the result was not statistically significant.

We have reported the first study to assess nutritional status and diet among CKD patients in the country. As with any observational study, there are a number of limitations to consider. Firstly this was a cross sectional study so we cannot comment on whether patients nutritional status changed over time. Secondly it was conducted at the only public run haemodialysis centre, and there are now private dialysis centres opening. Thirdly the staple diet is maize, and although maize is widely eaten in many African countries, other countries may have different dietary patterns. As with any observational study our findings should be interpreted with caution, as we can only report factors associated with malnourishment, but not apportion causality.

## Conclusion

There are differences in the population demographics of haemodialysis patients in economically advanced and those from developing countries. Despite a much younger patient cohort, with fewer co-morbidities, we found that malnutrition is common among CKD patients at the largest renal unit in the country, and higher serum albumin, creatinine, MUAC, body cell mass, and being employed were all protective against malnutrition.

## Data Availability

Primary data is held on a UCL server and in a MSc thesis held by UCL Library and data may be available on reasonable request, with all data de-identified in keeping with UK NHS practices.
